# Circadian Dependence of Infarct Size and Acute Heart Failure in ST Elevation Myocardial Infarction

**DOI:** 10.1371/journal.pone.0128526

**Published:** 2015-06-03

**Authors:** Aruni Seneviratna, Gek Hsiang Lim, Anju Devi, Leonardo P. Carvalho, Terrance Chua, Tian-Hai Koh, Huay-Cheem Tan, David Foo, Khim-Leng Tong, Hean-Yee Ong, A. Mark Richards, Chow Khuan Yew, Mark Y. Chan

**Affiliations:** 1 Cardiac Department, National University Heart Centre, National University Hospital, Singapore, Singapore; 2 National Registry of Diseases Office, (R&SP), Health Promotion Board, Singapore, Singapore; 3 Department of Medicine, Yong Loo Lin School of Medicine, National University of Singapore, Singapore, Singapore; 4 Hospital Israelita Albert Einstein, Sao Paolo, Brazil; 5 Department of Cardiology, National Heart Centre, Singapore, Singapore; 6 Department of Cardiology, Tan Tock Seng Hospital, Singapore, Singapore; 7 Department of Cardiology, Changi General Hospital, Singapore, Singapore; 8 Department of Cardiology, Khoo Teck Puat Hospital, Singapore, Singapore; University of Bologna, ITALY

## Abstract

**Objectives:**

There are conflicting data on the relationship between the time of symptom onset during the 24-hour cycle (circadian dependence) and infarct size in ST-elevation myocardial infarction (STEMI). Moreover, the impact of this circadian pattern of infarct size on clinical outcomes is unknown. We sought to study the circadian dependence of infarct size and its impact on clinical outcomes in STEMI.

**Methods:**

We studied 6,710 consecutive patients hospitalized for STEMI from 2006 to 2009 in a tropical climate with non-varying day-night cycles. We categorized the time of symptom onset into four 6-hour intervals: midnight–6:00 A.M., 6:00 A.M.–noon, noon–6:00 P.M. and 6:00 P.M.–midnight. We used peak creatine kinase as a surrogate marker of infarct size.

**Results:**

Midnight–6:00 A.M patients had the highest prevalence of diabetes mellitus (*P* = 0.03), more commonly presented with anterior MI (*P* = 0.03) and received percutaneous coronary intervention less frequently, as compared with other time intervals (*P* = 0.03). Adjusted mean peak creatine kinase was highest among midnight–6:00 A.M. patients and lowest among 6:00 A.M.–noon patients (2,590.8±2,839.1 IU/L and 2,336.3±2,386.6 IU/L, respectively, *P* = 0.04). Midnight–6:00 A.M patients were at greatest risk of acute heart failure (*P*<0.001), 30-day mortality (*P* = 0.03) and 1-year mortality (*P* = 0.03), while the converse was observed in 6:00 A.M.–noon patients. After adjusting for diabetes, infarct location and performance of percutaneous coronary intervention, circadian variations in acute heart failure incidence remained strongly significant (*P* = 0.001).

**Conclusion:**

We observed a circadian peak and nadir in infarct size during STEMI onset from midnight–6:00A.M and 6:00A.M.–noon respectively. The peak and nadir incidence of acute heart failure paralleled this circadian pattern. Differences in diabetes prevalence, infarct location and mechanical reperfusion may account partly for the observed circadian pattern of infarct size and acute heart failure.

## Introduction

Circadian rhythms are observed in many model systems of cardiovascular physiology and disease. Cardiovascular clinical events, such as myocardial ischemia [1–3], ventricular tachycardia [4] [5], sudden cardiac death [6] and stent thrombosis [7] frequently follow a circadian pattern with a peak incidence in the morning. Numerous studies have established a circadian pattern of acute myocardial infarction (AMI) onset with a peak incidence in the 6:00A.M.–noon interval [1–3, 6]. The higher risk of AMI occurring during this pre-waking time interval is attributed, among other factors, to increased sympathetic tone [8, 9], increased platelet aggregability [10, 11] and decreased plasma fibrinolytic activity [11, 12].

Beyond a circadian pattern of AMI incidence, recent studies have investigated the existence of a circadian pattern of ischemic tolerance and consequently, infarct size [13–16]. Animal studies suggest that a circadian pattern of ischemic tolerance is plausible, as infarcts induced in mice during different times in the 24-hour cycle can vary in size by as much as 3.5 fold [13]. In humans, cardiomyocyte clock genes may control diurnal variations in cardiomyocyte metabolic activity and may confer greater cardio protection at certain times within the 24-hour cycle, leading to circadian variability in ischemic tolerance [13]. These cardiomyocyte clock genes include PER1 and PER2, which peak in transcriptional activity in the morning, and BMAL1, which peaks in transcriptional activity in the evening [17].

A literature review revealed 4 population-based studies that had examined circadian patterns of infarct size in AMI. Two single center studies showed a peak infarct size among patients with midnight–6:00A.M. symptom onset [14, 15] while a third single center study showed a peak with 6:00A.M.–noon symptom onset [16]. A fourth study, the largest and only multicenter study (N = 1,099) to date, showed a numerical peak in infarct size with midnight–6:00A.M symptom onset that was not statistically significant [18]. All four studies had limited population sizes between 165 to 1099 patients, and none were large enough to examine the impact of circadian patterns of infarct size on clinical outcomes.

In this study, we examined circadian patterns of infarct size in a much larger prospective cohort of 6,710 consecutive patients with ST elevation myocardial infarction (STEMI) registered in the Singapore Myocardial Infarction Registry. We further investigated possible reasons accounting for circadian differences in infarct size, including differences in inhospital care during the 24-hour cycle. We then studied the relationship of circadian patterns in infarct size with clinical outcomes, including acute heart failure, left ventricular systolic dysfunction, arrhythmias, re-infarction, stroke and mortality. A key advantage of conducting a study on circadian patterns in Singapore is its tropical climate with non-varying day-night cycles and no seasonal fluctuations in temperature and light intensity.

## Methods

The Singapore Myocardial Infarction Registry (SMIR) is a nationwide registry of patients hospitalized for AMI [19]. The National Registry of Diseases Act was enacted to provide legislative cover for inclusion of personal healthcare information in selected national disease registries, including the Singapore Myocardial Infarction Registry, without the need for prior written informed consent [20]. This act allows for comprehensive disease coverage while ensuring privacy protection and data security. The study was approved by the National Healthcare Group Domain Specific Review Board with a waiver of written informed consent granted for research involving analysis of anonymised registry data.

Patients were identified through periodic reviews of hospital discharge diagnoses, laboratory records of cardiac enzyme measurements, death records from the registry of births and deaths, coroner postmortem reports and insurance claims data, as previously described [21]. Demographic data, co-morbidities, risk factors, in-hospital complications of AMI and left ventricular ejection fraction were extracted from patient hospital records by trained registry coordinators. Eight-hourly creatine phosphokinase (CK) and creatine phosphokinase-MB isoenzyme (CK-MB), troponin I and troponin T measurements were extracted from electronic laboratory records and the maximum among all eight-hourly values was considered the peak value. CK measurements have shown good correlation with infarct size measured using magnetic resonance imaging (MRI) [22] and single-photon emission computed tomography (SPECT) [23]. Peak CK was also used as the primary outcome measure in prior studies [14–16, 18]. We therefore selected peak CK as our surrogate marker of infarct size.

AMI cases were defined using International Classification of Diseases 9th Revision (ICD-9 Clinical Modification) code of 410 [24]. STEMI was defined as follows: typical chest pain of 30 min and significant ST segment elevation (0.1 mV or 0.2 mV on 2 adjacent limb or precordial leads, respectively, or new left bundle-branch block) and confirmed subsequently by a rise in biomarkers. Based on the culprit coronary artery, STEMI location was divided into anterior wall (left anterior descending coronary artery occlusion) or non-anterior wall (left circumflex or right coronary artery occlusion). All electrocardiograms were interpreted and all diagnoses were adjudicated centrally at the National Registry of Diseases Office. Self-reported time of onset of one or more symptoms—chest pain, breathlessness, diaphoresis, syncope, back pain, epigastric pain, jaw pain, shoulder pain—was captured as a continuous variable. The time of symptom onset was ascertained by the attending physician in the emergency department and recorded in the clinical notes.

Ischemic time was measured from the time of symptom onset to the time of successful deployment of the first device used to achieve reperfusion. Door-to-balloon time was measured from the time of arrival at the emergency department to the time of successful deployment of the first device used to achieve reperfusion.

Vital status was evaluated through record linkage with the National Registry of Births and Deaths to determine overall mortality. It is a statutory requirement that death be registered within 24 hours of its occurrence in Singapore. Previous studies have documented this information source to be complete [25]. Non-fatal outcomes were ascertained through review of hospital records on a quarterly basis. Re-infarction was defined as re-admission for a second AMI within 30 days of the index event. Follow up was complete for all patients in this study.

## Statistical Analysis

We divided time of symptom onset into four 6-hour intervals: midnight–6:00A.M., 6:00A.M.–noon, noon–6:00P.M. and 6:00P.M.–midnight, in accordance with previous reports [1, 2, 16, 25]. Data are expressed as mean ± SD for continuous variables that are normally distributed, or median (range) for skewed data. Percentages are presented for categorical variables. All comparisons between baseline variables were assessed using the Chi-square test for categorical variables and the analysis of variance (ANOVA) for parametric continuous variables or the Wilcoxon Rank-Sum test for non-parametric continuous variables. Mean infarct size across the 4 time intervals were compared using the ANOVA test and a Bonferroni correction was made for multiple comparisons across the 4 time intervals. In univariate analyses, we observed significant differences in the prevalence of diabetes, infarct location (anterior vs. inferior STEMI) and inhospital revascularization (inhospital revascularization vs. no inhospital revascularization) between the 4 time intervals. As such, we further adjusted for these as independent variables in the ANOVA model of infarct size. We plotted the relationship between mean peak log CK levels (surrogate of infarct size) and time of symptom onset using b-splines with 3 internal knots (turning points) at 3:00AM– 6:00AM, 6:00AM– 9:00AM and 6:00PM—9:00PM after centering on mean log peak CK. [26]. B-splines are piecewise polynomial functions that can be used to describe the shape of the data between any 2 knots. Due to the flexibility in the spline curve, it is able to assess any potential non-linear relationship between the exposure (time of symptom onset) and outcome of interest (infarct size). The incidence of secondary events, including left ventricular systolic dysfunction, acute heart failure, re-infarction, 30-day and 1-year mortality, were compared using Kaplan-Meier estimates. A multivariable logistic regression model was used to determine if time of symptom onset was an independent predictor of these secondary events. Independent variables identified from initial univariate analyses–diabetes, infarct location, inhospital revascularization and age- were included in this regression model for secondary events. For all analyses, *P*<0.05 was considered statistically significant. All statistical analysis was performed using STATA 11.0 (College Station, TX).

## Results

**Fig 1:**
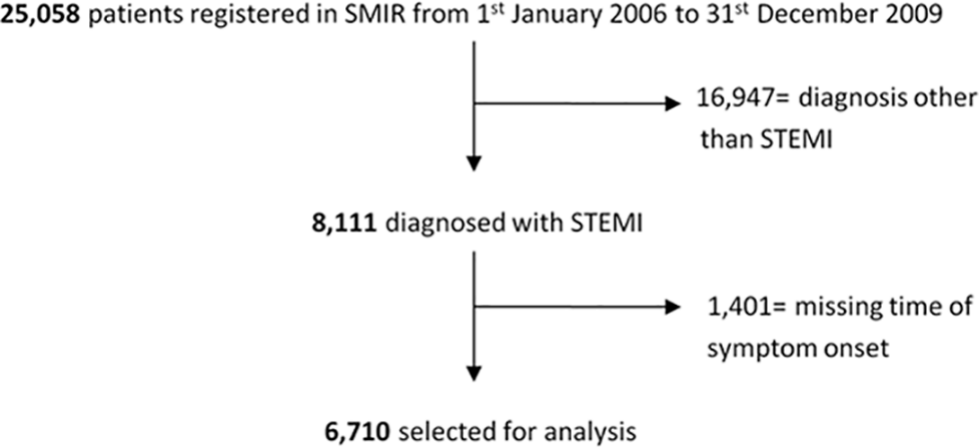
Study Flow. Of 25,058 patients registered in the Singapore Myocardial Infarction Registry (SMIR), 6,710 who were diagnosed with STEMI and with known times of symptom onset were selected for analysis.

From 1^st^ January 2006 to 31^st^ December 2009, 25,058 patients were registered in the Singapore Myocardial Infarct Registry, out of which 8,111 were diagnosed with STEMI. 1,401 patients (17.2%) with missing time of symptom onset were excluded from the final analysis (Fig 1).

### Differences in baseline characteristics in relation to time of symptom onset

Table 1 shows demographics and clinical characteristics categorized according to the time of symptom onset. The mean age of all patients in our study was 60.4 (standard deviation 13.0) years and 79.5% were males. The study population comprised of 64.1% Chinese, 18.9% Malay, 15.1% Indian and 1.9% other races. This racial distribution was comparable with the national ethnic composition of 74.1% Chinese, 13.4% Malay, 9.2% Indian and 3.3% other races [27], except for Indians, who had a relatively higher representation in the AMI cohort.

**Table 1:** Baseline characteristics of patients.

Time of symptom onset	Midnight–6:00 A.M.		6:00 A.M.–Noon		Noon–6:00 P.M.		6:00 P.M.–Midnight		P-value^a^
Number of cases (%)	1429	(21.3)	2102	(31.3)	1572	(23.4)	1607	(24.0)	
Age (years): Mean (SD)	60.7	(12.9)	60.7	(13.1)	60.3	(13.3)	59.7	(12.9)	0.99
Male (%)	1153	(80.7)	1651	(78.5)	1239	(78.8)	1290	(80.3)	0.99
Chinese (%)	887	(62.1)	1389	(66.1)	1032	(65.7)	992	(61.7)	0.37
Malay (%)	302	(21.1)	350	(16.7)	284	(18.1)	335	(20.9)	
Indian (%)	212	(14.8)	329	(15.7)	230	(14.6)	244	(15.2)	
Other (%)	28	(2.0)	34	(1.6)	26	(1.7)	36	(2.2)	
Current smoker (%)	630	(44.1)	823	(39.2)	691	(44.0)	715	(44.5)	0.04
Hypertension (%)	873	(61.1)	1200	(57.1)	869	(55.3)	926	(57.6)	0.21
Diabetes mellitus (%)	627	(43.9)	799	(38.0)	611	(38.9)	690	(42.9)	0.03
Dyslipidemia (%)	1076	(75.3)	1600	(76.1)	1167	(74.2)	1210	(75.3)	0.99
Past MI (%)	204	(14.3)	252	(12.0)	174	(11.1)	211	(13.1)	0.83
Weekend admission (%)	396	(27.7)	599	(28.5)	469	(29.8)	522	(32.4)	0.41
Anterior STEMI (%)	790	(55.3)	1064	(50.6)	819	(52.1)	857	(53.3)	0.03
Peak CK concentration (IU/L): mean (SD)	2590.8	(2839.1)	2336.3	(2386.6)	2526.8	(2809.7)	2522.1	(2645.2)	0.03

a With Bonferroni correction.

Baseline characteristics of patients categorized according to time of symptom onset. Data expressed as mean ± SD for continuous variables, median (range) for skewed data and percentages for categorical variables. Chi-square test was performed for categorical variables, analysis of variance for parametric continuous variables, Wilcoxon Rank-Sum test for non-parametric continuous variables.

Only 38% of 6:00A.M.–noon symptom onset patients had diabetes compared with 43.9% patients with midnight–6:00A.M. symptom onset (*P* = 0.03). Anterior STEMI was most prevalent among patients with midnight–6:00A.M. symptom onset (55.3%) and least prevalent among patients with 6:00A.M.–noon symptom onset (50.6%) (*P* = 0.03). There were fewer current smokers among patients with 6:00 A.M.-noon symptom onset (39.2%) as compared with the average frequency of smokers from the other 3 time intervals (44.2%). There was no significant difference in age, sex, race or the prevalence of hypertension, dyslipidemia or prior myocardial infarction.

### Treatment differences in relation to time of symptom onset

As shown in Table 2, Percutaneous Coronary Intervention (PCI) was performed on 73.3% of 6:00A.M.–noon symptom onset cases compared to 67.3% of cases with onset during other times of the day (*P* = 0.03). Patients with midnight-6:00A.M. symptom onset were 1.5 times more likely to undergo coronary artery bypass graft surgery (CABG) than patients with onset during other times of the day (*P* = 0.03), although, expectedly, overall rates of CABG were low in this STEMI cohort.

**Table 2:** Treatment differences among different time intervals.

Time of symptom onset	Midnight–6:00A.M.		6:00A.M.–Noon		Noon–6:00P.M.		6:00P.M.–Midnight		P-value
**Medical treatment (in hospital)**									
Aspirin (%)	1229	(86.7)	1859	(89.0)	1369	(87.9)	1408	(88.4)	0.99
P2Y_12_ antagonist (%)	1221	(86.2)	1844	(88.3)	1357	(87.2)	1402	(88.1)	0.99
Beta-blocker (%)	1086	(76.6)	1629	(78.0)	1190	(76.4)	1233	(77.5)	0.99
ACE/ARB (%)	998	(70.4)	1451	(69.5)	1034	(66.4)	1142	(71.7)	0.34
Lipid lowering therapy/statin (%)	1240	(87.5)	1876	(89.9)	1378	(88.5)	1416	(88.9)	0.99
**Medical treatment (administered within 24 hours of admission)**									
Aspirin (%)	1193	(83.5)	1821	(86.6)	1343	(85.5)	1376	(85.7)	0.99
P2Y_12_ antagonist (%)	1186	(83.1)	1790	(85.2)	1313	(83.6)	1352	(84.2)	0.99
Beta-blocker (%)	622	(43.6)	1005	(47.8)	715	(45.5)	745	(46.4)	0.99
**Invasive management (in hospital)**									
Door-to-balloon time: Mean (SD^a^), hours	1.8	(1.2)	1.6	(1.0)	1.6	(0.9)	1.8	(1.1)	0.07
Door-to-balloon time: Median (range), hours	1.5	(0.1–11.0)	1.4	(0.3–11.6)	1.4	(0.2–11.7)	1.5	(0.4–10.0)	0.07
Ischemic time (hours): mean (SD)	5.9	(5.3)	4.6	(3.8)	4.4	(4.0)	5.9	(6.1)	0.68
Ischemic time (hours): median (range)	4.4	(0.9–75.6)	3.6	(0.4–33.4)	3.3	(0.3–37.2)	3.7	(0.6–71.9)	0.68
PCI^b^ (%)	956	(67.0)	1540	(73.3)	1089	(69.3)	1053	(65.6)	0.03
CABG^c^ (%)	48	(3.4)	53	(2.5)	37	(2.4)	45	(2.8)	0.03

a SD = standard deviation

b PCI = Percutaneous coronary intervention

c CABG = coronary artery bypass grafting

Differences in revascularization treatments received by patients categorized according to time of symptom onset. Ischemic and door-to-balloon times were measured from the time of symptom onset and the time of arrival at the emergency department to the time of first device deployment respectively.

Door-to-balloon times were longer after regular working hours (6:00 P.M.–6:00 A.M.) than during regular working hours (6:00 A.M.–6:00 PM), but this difference in door-to-balloon times did not reach statistical significance (*P* = 0.07). Mean ischemic time was longest among patients with midnight–6:00A.M. symptom onset, although this difference did not reach statistical significance (*P* = 0.68). We did not observe differences in the use of aspirin, P2Y_12_ antagonists, beta-blockers, ACE inhibitors, angiotensin receptor blockers, statins or other lipid lowering therapy among the four time intervals (Table 2).

### Circadian pattern of STEMI incidence and infarct size

**Fig 2:**
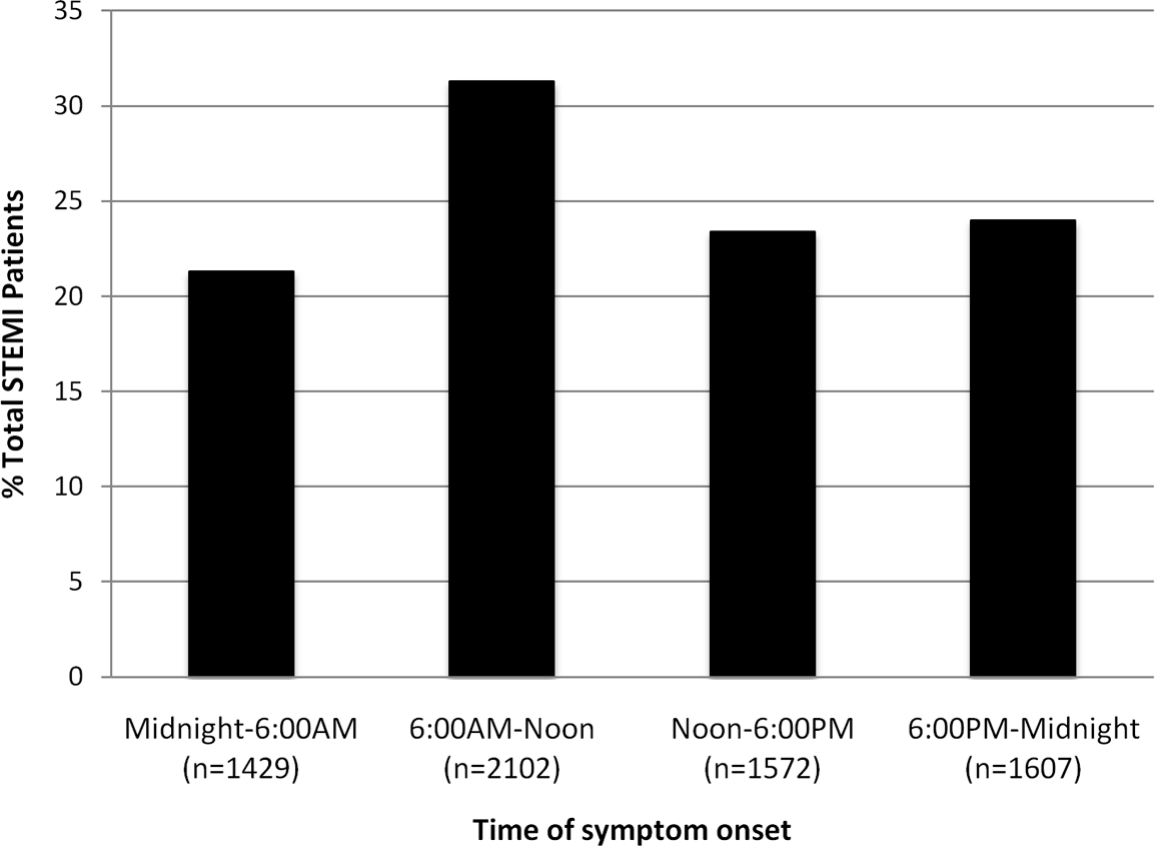
STEMI Incidence as a function of time of symptom onset. Bar chart showing number of patients with symptom onset within each of the 4 pre specified time intervals. The peak incidence of symptom onset was observed in the 6:00-noon period (*P*<0.001).

The peak incidence of symptom onset was observed during the 6:00A.M.–noon interval (N = 2102, 31.3%), an observation consistent with the majority of prior studies, while the lowest incidence of symptom onset was observed during the midnight–6:00A.M. interval (N = 1429, 21.3%) (Fig 2).

**Fig 3:**
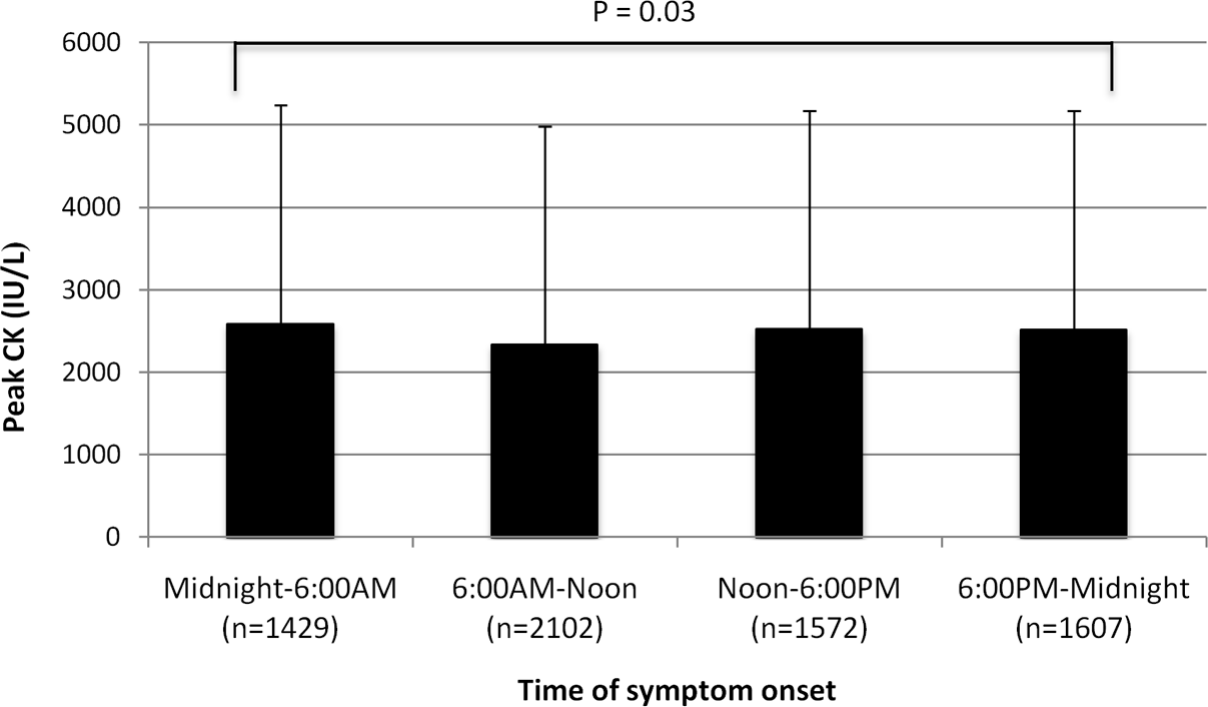
Infarct size as a function of time of symptom onset. Mean Peak log creatine kinase (CK) concentration of patients with symptom onset within each of the 4 time intervals. Wide bars represent mean concentration and T bars represent standard deviation (SD). The maximum and minimum mean peak logCK concentration was observed with symptom onset from midnight-6:00 A.M. and 6:00 A.M.-noon respectively. Comparison of mean peak logCK across 4 time intervals was performed using the Kruskall-Wallis test followed by a Bonferroni correction (*P* = 0.03). The difference remained statistically significant after adjusting for the presence of diabetes, infarct location and use of PCI (*P* = 0.04).

The highest mean peak CK values were observed among patients with midnight–6:00A.M. symptom onset (2590.8, SD 2839.1 IU/L) and lowest values among patients with 6:00A.M.–noon symptom onset (2336.3, SD 2386.6 IU/L, *P* = 0.03) (Fig 3). Patients with 6:00A.M.–noon symptom onset had 8–10% smaller mean infarct sizes than the remaining three time intervals. Distribution of peak CK concentration with respect to onset time is further illustrated in Figs A and B in S1 File. The distribution of infarct size by time of symptom onset (Table A in S1 File) and time of arrival at hospital (Table B in S1 File) on a finer granularity of every 3 hours showed a pattern that was consistent with that seen in the analysis using 6-hour time intervals. In multivariable analysis, after adjusting for differences in diabetes prevalence, infarct location and PCI rates, differences in infarct size remained statistically significant (*P* = 0.04).

**Fig 4:**
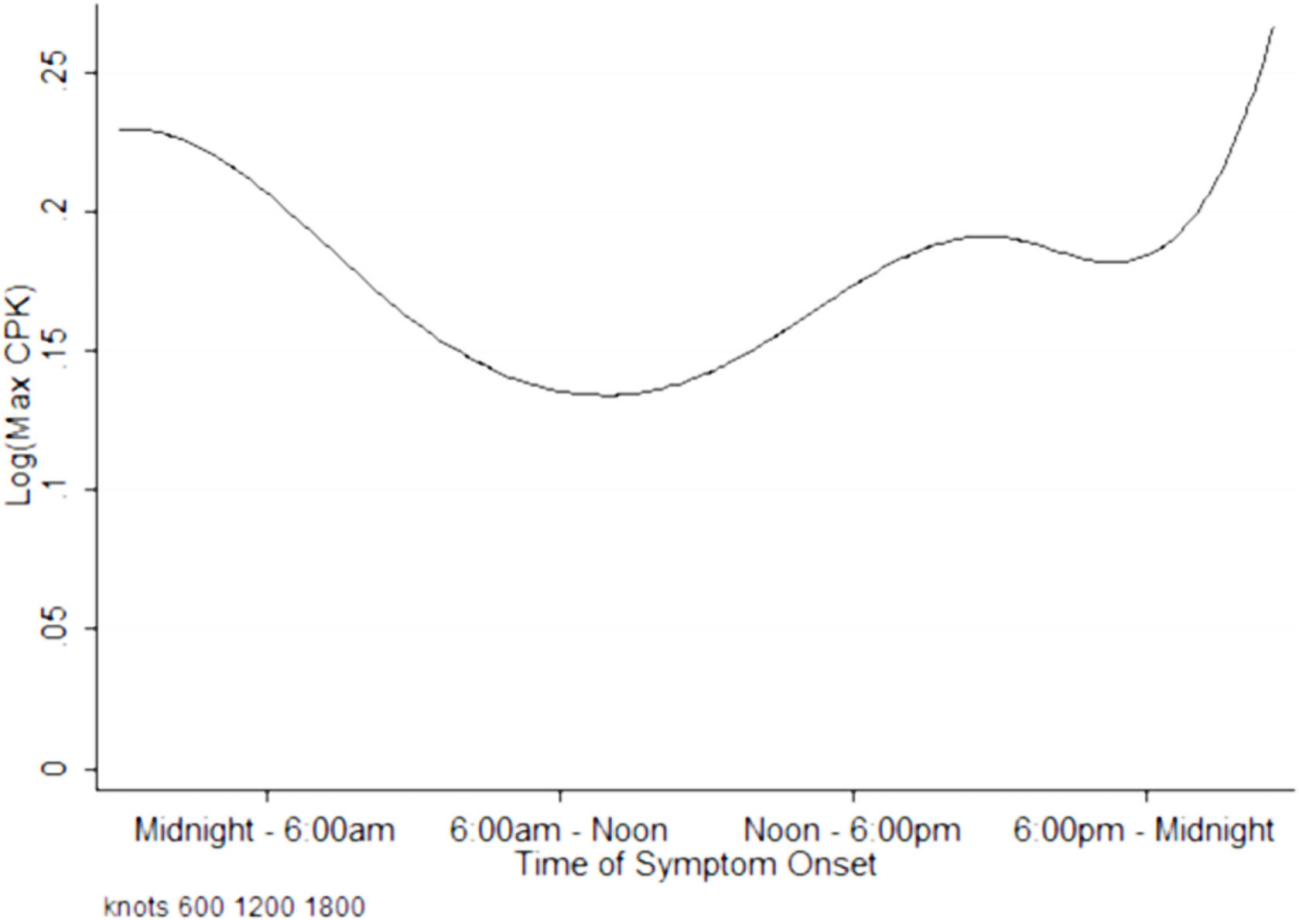
Fitted curve of Peak concentration of log (CK) against time of symptom onset. Non-linear B-splines with 3 internal knots (turning points) between 3:00AM– 6:00AM, 6:00AM– 9:00AM and 6:00PM—9:00PM, centering on mean log peak CK, to assess any potential non-linear relationship between the exposure (time of symptom onset) and outcome of interest (infarct size).

We then fitted restricted cubic splines to describe the shape of the association between time of symptom onset and myocardial infarct size. Restricted cubic splines of log maximum CK against time of symptom onset showed three turning points at 3:00AM– 6:00AM, 6:00AM– 9:00AM and 6:00PM—9:00PM (Fig 4). The highest peak was observed among patients with symptom onset within the midnight–6:00A.M. interval, while the lowest nadir was observed among patients with symptom onset within the 6:00A.M.–noon interval. However, the test for non-linearity using restricted cubic splines was non-significant (*P* = 0.07), and while there were differences in categories of symptom onset time, chance findings in the spline association between time of symptom onset and infarct size could not be excluded.

### Circadian dependence of clinical outcomes

Patients with midnight–6:00A.M. symptom onset were at a 30% higher relative risk of developing acute heart failure. As shown in Table 3, 22.8% of midnight–6:00A.M. cases presented with Killip class II-IV (*P*<0.001) in contrast to only 15.5% of 6:00A.M.–noon cases. Older age (odds ratio [OR] 1.04 per year increase in age, *P*<0.001), diabetes (OR 1.45, *P<*0.001), prior AMI (OR 1.86, *P<*0.001) and anterior infarct (OR 2.35, *P*<0.001) were independently associated with the occurrence of acute heart failure.

**Table 3:** Complications of AMI and clinical outcomes.

Time of symptom onset	Midnight–6:00A.M.		6:00A.M.–Noon		Noon–6:00P.M.		6:00P.M.–Midnight		P-value
LVEF (%)	35.0%±8.7		37.0±8.0		36.5±8.7		35.6±8.5		0.03
Left ventricular systolic dysfunction (%)^a^	829	(58.6)	1081	(52.0)	847	(54.5)	948	(59.7)	0.03
Acute heart failure (%)^b^	326	(22.8)	326	(15.5)	271	(17.2)	334	(20.8)	<0.001
Cardiogenic shock (%)	140	(15.4)	149	(11.8)	138	(13.8)	135	(13.5)	0.99
Atrial Fibrillation (%)	105	(7.4)	127	(6.0)	118	(7.5)	104	(6.5)	0.03
VF/sustained VT (%)	91	(6.4)	108	(5.1)	101	(6.4)	109	(6.8)	0.99
Complete heart block (%)	49	(3.5)	77	(3.7)	60	(3.9)	70	(4.4)	0.99
Acute renal failure (%)	92	(6.5)	104	(5.0)	91	(5.9)	90	(5.7)	0.99
Cerebrovascular accident (%)	22	(1.6)	21	(1.0)	25	(1.6)	29	(1.8)	0.99
1-year mortality (%)	298	(20.9)	346	(16.5)	292	(18.6)	276	(17.2)	0.03
30-day mortality (%)	237	(16.6)	274	(13.0)	241	(15.3)	220	(13.7)	0.03
Reinfarction (%)	25	(2.8)	27	(2.1)	18	(1.8)	25	(2.5)	0.78

a Left ventricular systolic dysfunction defined as LVEF < 50% on echocardiography

b Acute heart failure defined as Killip class ≥ II.

Short- and long-term clinical outcomes categorized according to time of symptom onset.

The worst left ventricular ejection fraction was observed among patients with midnight–6:00A.M. symptom onset (35.0% SD 8.7%, *P* = 0.03) (Table 3). Left ventricular systolic dysfunction, defined as left ventricular ejection fraction <50%, was observed in 58.6% of patients with midnight–6:00A.M. symptom onset compared with 52.0% of patients with 6:00A.M.–noon symptom onset (*P* = 0.03). Prior myocardial infarction (OR 2.29, *P<*0.001), longer ischemic times (OR 1.06, *P<*0.001) and anterior location of infarct (OR 3.79, *P<*0.001) were independently associated with the development of left ventricular systolic dysfunction. Table 3 shows that atrial fibrillation occurred least commonly among patients with 6:00 A.M.–noon symptom onset (*P* = 0.03), which was also the nadir of infarct size. We did not observe any difference in development of ventricular fibrillation or complete heart block among the four time intervals.

**Fig 5:**
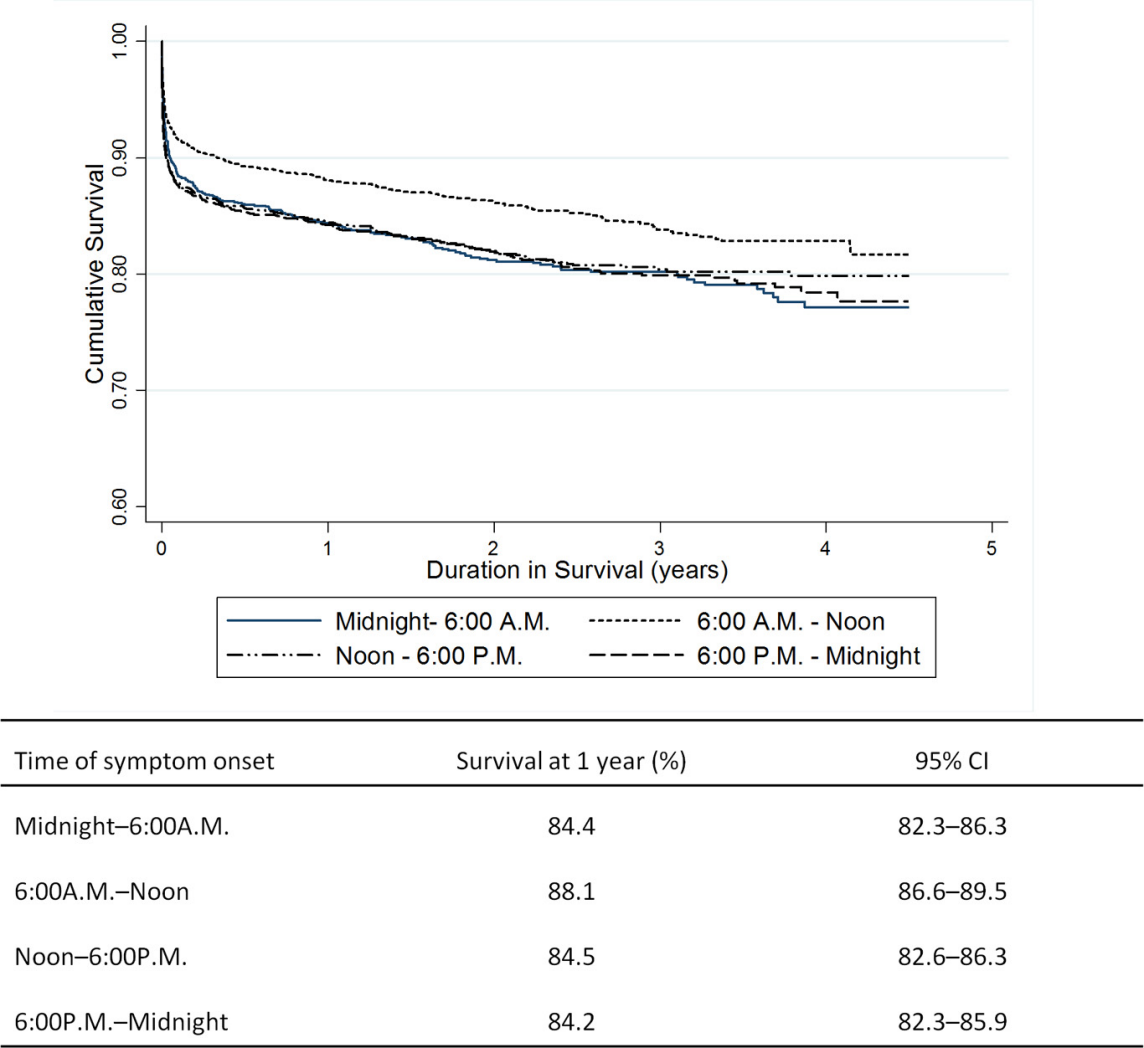
Long-term survival as a function of time of symptom onset. Survival curves out to 4.5 years of follow up shown above and Kaplan-Meier estimates of 12-month survival shown below.

Unadjusted Kaplan-Meier estimates of 1-year survival for each of the four time intervals showed that patients with 6:00A.M.–noon symptom onset had significantly better survival than the other three time intervals (Fig 5). This improved survival was sustained during 4 years of follow-up. Patients with 6:00A.M.–noon symptom onset had an absolute 1-year mortality rate 4% lower than those in the remaining three time intervals (Fig 5).

After adjusting for diabetes prevalence, infarct location (anterior vs. non-anterior) and performance of percutaneous coronary intervention in multivariable analysis (Table 4), patients with 6:00 A.M.–noon symptom onset remained at the lowest risk of acute heart failure, with a 31% lower relative odds of developing heart failure compared to patients with midnight–6:00A.M. symptom onset (*P* = 0.001). Statistical significance was no longer observed in circadian patterns of left ventricular systolic dysfunction (*P* = 0.07), reinfarction (*P* = 0.85), 30-day mortality (*P* = 0.47) and 1-year mortality (*P* = 0.70) after multivariable adjustment.

**Table 4:** Multivariable logistic regression analysis of clinical outcomes.

Time of symptom onset	1-year mortality		30-day mortality		Reinfarction		Left ventricular systolic dysfunction		Killip Class (I vs. II—IV)	
	OR (95% CI)	P-value	OR (95% CI)	P-value	OR (95% CI)	P-value	OR (95% CI)	P-value	OR (95% CI)	P-value
Midnight–6:00 A.M.	1 (reference group)	0.7	1 (reference group)	0.47	1 (reference group)	0.85	1 (reference group)	0.07	1 (reference group)	0.001
6:00 A.M.–Noon	0.73 (0.35–1.49)		0.93 (0.53–1.64)		1.16 (0.50–2.70)		0.83 (0.67–1.02)		0.69 (0.57–0.82)	
Noon–6:00 P.M.	1.07 (0.47–2.44)		1.47 (0.79–2.75)		0.81 (0.30–2.21)		1.05 (0.83–1.33)		0.73 (0.61–0.88)	
6:00 P.M.–Midnight	0.77 (0.35–1.71)		1.20 (0.64–2.23)		1.24 (0.50–3.05)		1.02 (0.80–1.29)		0.88 (0.74–1.05)	

A multivariable logistic regression model adjusting for diabetes prevalence, infarct location (anterior vs. non-anterior) and performance of percutaneous coronary intervention (PCI) was used to determine if time of symptom onset was an independent predictor of secondary outcomes.

## Discussion

We demonstrated a circadian pattern of MI incidence, infarct size and clinical outcomes in STEMI. Midnight-6:00 A.M. symptom onset was associated with lowest MI incidence, the largest mean infarct size and the worst clinical outcomes, while 6:00 A.M.–noon symptom onset was associated with the highest MI incidence, the smallest mean infarct size and the best clinical outcomes. Unlike prior studies on circadian patterns of infarct size, our study was conducted under tropical climate conditions with non-varying day-night cycles and no seasonal fluctuations in temperature and light intensity.

With more than 6,700 patients with STEMI, our study represents, to the best of our knowledge, the largest analysis of circadian patterns of infarct size. Of the 4 known prior studies on circadian patterns of infarct size in STEMI (13, 14, 15 and 17), the results of 3 studies (13, 14 and 17) concur with ours. A multicenter study of 1,099 patients, the second largest study after ours, showed that the mean infarct size was numerically largest (peak) during midnight-6:00AM symptom onset and smallest (nadir) during 6:00A.M.–noon symptom onset, although their results did not reach statistical significance [18]. In our study, in which statistical significance was achieved, each of the four 6-hour time intervals alone had more than 1,400 patients. Two other smaller studies with total study population sizes of 165 and 353 patients also showed a statistically significant nadir and peak in infarct size with 6:00A.M.–noon symptom onset and midnight-6:00AM symptom onset respectively [14, 15]. Of the five known studies to date investigating circadian patterns of infarct size, including ours, only one study by Saurez-Barrientos et al. [16] showed a peak infarct size with 6:00A.M.–noon onset and nadir infarct size with noon–6:00P.M. onset, which was essentially a rightward shift of the circadian pattern by one 6-hour time interval compared with the circadian pattern in all other studies (Table C in S1 File).

Multiple factors influence the ultimate infarct size following prolonged ischemia. Variations in the accessibility and quality of prehospital and hospital care during different times of the day are frequently implicated factors. Studies across multiple healthcare systems have shown that delays in emergency service mobilization and hospital treatment occur more frequently after regular working hours, the so-called ‘after-hours’ effect [28, 29]. Longer time to treatment has been shown to be linearly associated with infarct size in STEMI [30]. Higher rates of angioplasty failure and in-hospital mortality are also observed when patients with STEMI are managed ‘after-hours’ as compared with regular working hours [31]. Although differences in door-to-balloon time and PCI rates across the 4 time intervals did not reach statistical significant in our study, it is conceivable that differential access to care and type of treatment may contribute to the better outcomes of patients with symptom onset during the 6:00A.M.–noon interval.

Our study revealed other potential explanations for the observed circadian patterns in infarct size. We observed a significantly higher prevalence of diabetes with midnight–6:00A.M. symptom onset. Diabetic patients may be at greater risk of MI during sleep because of cardiovascular autonomic neuropathy, which causes impaired perception of ischemic pain leading to delay in recognition, diagnosis and treatment of an ongoing myocardial infarction [32]. Diabetic patients have been observed to suffer larger infarcts with the same degree of ischemia [33].

We further observed that anterior wall infarcts were most common among patients with midnight–6:00A.M. symptom onset and least common among patients with 6:00A.M.–noon symptom onset. This likely accounted in part for the larger and smaller infarct sizes during the midnight–6:00A.M. and 6:00A.M.–noon intervals, respectively. Two other studies, also conducted in Asian cohorts, have shown higher incidence of anterior infarcts with midnight–6:00A.M.onset [34, 35], but further studies are needed to understand pathophysiological mechanisms underlying infarct location during the 24-hour cycle.

Consistent with other studies [3, 12, 16, 18, 36, 37], we observed the highest incidence in MI during 6:00A.M.–noon interval. Higher risk of myocardial ischemia in morning hours is attributed to a combination of neurohumoral and cardiac factors. These include increased sympathetic tone [8], increased vascular resistance, increased platelet aggregability [10, 11], decrease in plasma fibrinolytic activity [12] and enhanced morning cortisol awakening response [38]. Moreover, Durgan et al. have identified cardiomyocyte clock genes that mediate time-of-day dependence in myocardial tolerance to ischemia [13] and thereby influence infarct size by modulating the level of ischemic tolerance throughout the 24-hour cycle. Yet, discriminating the relative influence of intrinsic and environmental factors remains challenging in human studies.

In our study, circadian patterns of infarct size closely paralleled circadian patterns of clinical outcomes, including left ventricular systolic dysfunction, acute heart failure, new onset atrial fibrillation, 30-day and 1-year mortality after STEMI. The most robust circadian pattern of clinical outcomes was observed with acute heart failure. Even after adjustment for all other possible factors influencing the development of acute heart failure during AMI, we observed a circadian peak and nadir in acute heart failure events among patients with midnight–6:00A.M. and 6:00A.M.–noon symptom onset, corresponding to the circadian peak and nadir in infarct size, respectively. Our findings are consistent with those of Mukamal et al. [39] in which the highest risk of congestive heart failure was observed among infarctions with early morning symptom onset. While one might speculate that circadian differences in infarct size led to these circadian differences in acute heart failure, it is uncertain whether the absolute differences of 8–10% in infarct size between time intervals could have accounted for these circadian differences in the incidence of acute heart failure.

Our study has several limitations that deserve comment. First, infarct size was ascertained using peak CK measurements whereas newer imaging techniques, such as MRI and SPECT, are expected to deliver greater sensitivity and specificity than biochemical determination of infarct size. However, all four prior studies have used peak CK as their primary outcome measure [14–16, 18] and this method has shown good correlation with infarct size measured using MRI [22] and SPECT [23]. Second, the success of reperfusion is an important determinant of infarct size [40] and clinical outcomes [41]. As angiographic data are not captured in the Singapore Myocardial Infarct Registry, we are unable to comment on the success of reperfusion therapy and its impact on clinical outcomes. In particular, we were unable to establish the presence of complete occlusion (TIMI 0 flow) or collateral coronary blood flow as angiographic determinants of infarct size. Third, our multicenter study, like that of Ammirati et al. [18], is expected to introduce greater variability in estimates of infarct size, as compared with single-center studies. Nonetheless, a central team of coordinators at the SMIR abstracted data using a standardized case report form and all clinical outcomes were centrally adjudicated following a standard protocol. Fourth, as for all human studies that require information on the time of symptom onset in STEMI, survivor bias cannot be totally eliminated as the data are inherently limited to patients who survive till the time of first medical contact. Lastly, the test for non-linearity using restricted cubic splines was non-significant (*P* = 0.07), chance findings in the spline association between time of symptom onset and infarct size could not be excluded. However, the robustness of the association between categories of time of symptom onset and infarct size was preserved even after further subdivision of categories of time of symptom onset into 3-hour windows and multivariable adjustment for significant independent variables including diabetes prevalence, infarct location (anterior versus inferior STEMI) and performance of primary PCI.

The strengths of our study include a study population six-times larger than any other similar study, with a sufficient number of clinical events within each 6-hour time interval to relate circadian patterns of infarct size with clinical outcomes, and non-varying day-night cycles with no seasonal fluctuations in temperature and light intensity.

## Conclusion

We observed a circadian dependence of infarct size and acute heart failure outcomes among patients hospitalized for STEMI. Symptom onset during the 6:00 A.M.–noon interval was associated with the highest incidence of MI and the smallest mean infarct size (nadir) while the opposite was observed with symptom onset during the midnight-6:00 A.M. interval. The 6:00 A.M-noon nadir and midnight-6:00 A.M peak in infarct size largely corroborate with the result of prior similar studies. Because we had a study population that was six-fold greater than prior similar studies, we were able to further establish a circadian relationship between infarct size and acute heart failure, in which acute heart failure occurred least frequently during the 6:00 A.M-noon nadir in infarct size and most frequently during the midnight-6:00 A.M peak in infarct size. Interestingly, patients with diabetes appear to be at higher risk of STEMI onset during midnight–6:00A.M. Our study further suggests that circadian differences in diabetes prevalence, infarct location and mechanical reperfusion may account partly for the observed circadian pattern of infarct size and acute heart failure.

## Supporting Information

S1 File Table A- Infarct size as a function of time of symptom onset. The distribution of infarct size (peak CK) by time of symptom onset on a finer granularity of every 3 hours. **Table B-** Infarct size as a function of time of arrival at hospital. The distribution of infarct size (peak CK) by time of arrival at hospital on a finer granularity of every 3 hours. **Table C-** Human studies on circadian dependence of infarct size. **Fig A-** Histograms to show distribution of peak creatine kinase (CK) concentration with respect to time of symptom onset. **Fig B-** Scatterplot to show distribution of peak creatine kinase (CK) concentration with respect to time of symptom onset.
(DOCX)
