# Supplementary material for: Circadian Dependence of Infarct Size and Acute Heart Failure in ST Elevation Myocardial Infarction
Source: PLoS One. 2015 Jun 3;10(6):e0128526. doi: 10.1371/journal.pone.0128526 (PMC4454698; doi:10.1371/journal.pone.0128526)
Supplement: S1 File — The distribution of infarct size (peak CK) by time of symptom onset on a finer granularity of every 3 hours. Table B- Infarct size as a function of time of arrival at hospital. The distribution of infarct size (peak CK) by time of arrival at hospital on a finer granularity of every 3 hours. Table C- Human studies on circadian dependence of infarct size. Fig A- Histograms to show distribution of peak creatine kinase (CK) concentration with respect to time of symptom onset. Fig B- Scatterplot to show distribution of peak creatine kinase (CK) concentration with respect to time of symptom onset. (DOCX) [file pone.0128526.s001.docx]

**S1 File**

**Table A-** Infarct size as a function of time of symptom onset

| Time of symptom onset | Midnight – 3:00 AM | | 3:00AM – 6:00AM | | 6:00AM – 9:00AM | | 9:00AM – Noon | | Noon – 3:00PM | | 3:00PM – 6:00PM | | 6:00PM – 9:00PM | | 9:00PM – Midnight | |
| --- | --- | --- | --- | --- | --- | --- | --- | --- | --- | --- | --- | --- | --- | --- | --- | --- |
| Number of cases (%) | 884 | (13.1) | 713 | (10.6) | 1142 | (17.0) | 960 | (14.3) | 833 | (12.4) | 739 | (11.0) | 826 | (12.3) | 613 | (9.1) |
| Peak CK concentration (IU/L): mean (SD) | 2559.9 (2473.6) | | 2603.5 (3126.3) | | 2302.4 (2385.4) | | 2377.3 (2388.7) | | 2505.4 (2776.8) | | 2551.3 (2848.8) | | 2574.2 (2897.2) | | 2461.9 (2376.3) | |

**Table B-** Infarct size as a function of time of arrival at hospital

| Time of arrival at hospital | Midnight – 3:00 AM | | 3:00AM – 6:00AM | | 6:00AM – 9:00AM | | 9:00AM –  Noon | | Noon – 3:00PM | | 3:00PM – 6:00PM | | 6:00PM – 9:00PM | | 9:00PM – Midnight | |
| --- | --- | --- | --- | --- | --- | --- | --- | --- | --- | --- | --- | --- | --- | --- | --- | --- |
| Number of cases (%) | 555 | (9.2) | 414 | (6.9) | 607 | (10.1) | 1247 | (20.7) | 955 | (15.8) | 838 | (13.9) | 707 | (11.7) | 708 | (11.7) |
| Peak CK concentration (IU/L): mean (SD) | 2721.3 (2397.4) | | 2962.1 (2753.9) | | 2693.4 (2940.1) | | 2448.3  (2453.5) | | 2376.7 (2246.8) | | 2569.5  (3001.0) | | 2692.9 (2689.3) | | 2689.2  (2556.7) | |

The distribution of infarct size (peak CK) by time of symptom onset (S1 Table) and time of arrival at hospital (S2 Table) on a finer granularity of every 3 hours. Percentage of patients by the 3 hourly time of symptom onset categorization ranged from 9.1% to 17.0%. Correspondingly, the percentage of patients by time of arrival at hospital ranged from 9.2% to 20.7%.

**Table C-** Human studies on circadian dependence of infarct size

| Study | Country | Total no. (patients analyzed) | No. of hospitals | % primary PCI^a^ | Mean age, years | Mean ischemic time, min | Mean peak CK^b^, IU/L | Time of peak infarct size | Time of trough infarct size |
| --- | --- | --- | --- | --- | --- | --- | --- | --- | --- |
| Suarez-Barrientos [[16](file:///G:\Plos%20one%20circadian%20revision\PLOS%20one%20third%20review\Circadian_Rhythm_STEMI_PLOS%20One_MYC.docx#_ENREF_16)] | Spain | 950 (811) | 1 | 79 | 62 | 229 | 1600 | 6:00A.M.–noon | noon–6:00P.M. |
| Reiter [[14](file:///G:\Plos%20one%20circadian%20revision\PLOS%20one%20third%20review\Circadian_Rhythm_STEMI_PLOS%20One_MYC.docx#_ENREF_14)] | USA | 1031 (165) | 1 | 100 | 59 | 168 | 2543 | 1:00A.M. | ? noon^c^ |
| Fournier [[15](file:///G:\Plos%20one%20circadian%20revision\PLOS%20one%20third%20review\Circadian_Rhythm_STEMI_PLOS%20One_MYC.docx#_ENREF_15)] | Switzerland | 588 (353) | 1 | 100 | 66 | 162 | 2687 | midnight–6:00A.M. | 6:00A.M.–noon |
| Ammirati [[18](file:///G:\Plos%20one%20circadian%20revision\PLOS%20one%20third%20review\Circadian_Rhythm_STEMI_PLOS%20One_MYC.docx#_ENREF_18)] | China, Scotland and Italy | 1099 (1099) | 32 | 52 | 61 | 180 | 1606 | midnight–6:00A.M.  (not statistically significant) | 6:00A.M.–noon  (not statistically significant) |
| Seneviratna | Singapore | 8111 (6710) | 6 | 60.4 | 61 | 306 | 2271 | midnight–6:00A.M. | 6:00A.M.–noon |

^a^ CK = creatine kinase, ^b^ PCI = percutaneous coronary intervention

^c^ time of trough infarct size estimated from figure in study by Reiter et al (Circ res 2012;110:105-110)

Modified from Traverse, J. H. Circulation research 2013, e115

**Figure A-** Histograms to show distribution of peak creatine kinase (CK) concentration with respect to time of symptom onset.

0

2.0e-04

4.0e-04

6.0e-04

0

2.0e-04

4.0e-04

6.0e-04

0

2000

4000

6000

8000

0

2000

4000

6000

8000

Midnight–6:00 A.M.

6:00 A.M.–noon

Noon–6:00 P.M.

6:00 P.M.–midnight

Peak CK Concentration

Density

**Figure B-** Scatterplot to show distribution of peak creatine kinase (CK) concentration with respect to time of symptom onset.
